# A Delphi consensus for implementing genomic testing in unresectable or metastatic urothelial cancer

**DOI:** 10.1111/bju.70278

**Published:** 2026-04-27

**Authors:** Syed A. Hussain, Amarnath Challapalli, Simran Gill, Michael Hubank, Hannah Markham, Philippe Tanière, Simon Wyatt, Robert Jones

**Affiliations:** ^1^ Division of Clinical Medicine, School of Medicine and Population Health University of Sheffield and Sheffield Teaching Hospitals Sheffield UK; ^2^ Bristol Cancer Institute Bristol UK; ^3^ Johnson and Johnson Innovative Medicine High Wycombe UK; ^4^ Royal Marsden Hospital London UK; ^5^ University Hospital Southampton Southampton UK; ^6^ Queen Elizabeth Hospital Birmingham UK; ^7^ University of Glasgow Beatson West of Scotland Cancer Centre Glasgow UK

**Keywords:** genomic test, urothelial cancer, bladder cancer, Delphi consensus, UK

## Abstract

**Objectives:**

To establish a UK consensus vision of a standardised genomic testing pathway in unresectable/metastatic urothelial cancer (mUC) with clear roles and responsibilities for multidisciplinary team (MDT) members and molecular pathology laboratory teams, and to agree how the pathway must evolve to accommodate emerging innovations.

**Subjects and Methods:**

A modified Delphi method was used. Six healthcare professionals (HCPs) with expertise in UC formed the Steering Committee (SC), which met in May 2025. Seven key consensus topics were developed covering the steps of the pre‐testing and genomic testing pathways, the current and future state of precision medicine, and approaches to overcome barriers and ensure readiness for the future. Within these topics, statements were developed and distributed to clinical scientists and HCPs via an on‐line 4‐point Likert scale survey. Respondents could opt out of responding to statements outside their area of expertise. Consensus was pre‐defined as ≥75% agreement.

**Results:**

Overall, 50 responses were received; consensus was reached on 41/42 statements. The statement that did not achieve consensus was part of a paired set. A proposed pathway for pre‐testing and genomic testing in unresectable or mUC was developed based on survey results and discussions at SC meetings. Key priorities for the pathway include: timely fibroblast growth factor receptor 3 testing, ideally at the commencement of first‐line systemic therapies; selection of specimens based on volume of tissue and recency; immediate access to the genomic testing report for all MDT members; and secure data environments with federated access to genomic results. Since the stopping criteria were met, no further survey rounds were required.

**Conclusion:**

Adopting the genomic testing pathway derived from this Delphi consensus will provide greater clarity around MDT and molecular laboratory responsibilities, reduce regional inequities in access, and optimise patient outcomes through timely delivery of genomic testing and subsequent treatment.

**Abbreviations**
ACCORD: Accurate Consensus Reporting Document
AWMGS: All Wales Medical Genomics Service
CE: Conformité Européenne
CGLP: Current Good Laboratory Practice
DNA: deoxyribonucleic acid
EAU: European Association of Urology
ESMO: European Society for Medical Oncology
FGFR(2)(3): fibroblast growth factor receptor (2) (3)
GMS: Genomic Medicine Service
HCP: healthcare professional
J&J IM: Johnson and Johnson Innovative Medicine
MDT: multidisciplinary team
MHRA: Medicines and Healthcare products Regulatory Agency
mUC: metastatic urothelial cancer
NGS: next‐generation sequencing
NICE: National Institute for Health and Care Excellence
NTRK: neurotrophic tyrosine receptor kinase
PD‐1: programmed cell death protein 1
PD‐L1: programmed cell death ligand 1
QPI: quality performance indicator
RMDS: Regional Molecular Diagnostics Service
RNA: ribonucleic acid
S(number): statement
SC: Steering Committee
SSNGM: Scottish Strategic Network for Genomic Medicine
TURBT: transurethral resection of bladder tumour
UC: urothelial cancer
UKAS: United Kingdom Accreditation Service

## Introduction

Bladder cancer remains a substantial health burden with >18 000 new cases diagnosed in the UK in 2022 [1]. In the same year, of the cases with a known stage, 12% in England and 18% Scotland were metastatic (Stage IV) [1]. Some cases of locally advanced bladder cancer are also unresectable. The most common form of bladder cancer in the UK is urothelial, accounting for ~90% of cases [2].

For patients with unresectable or metastatic urothelial cancer (mUC), current treatment options include chemotherapy, programmed cell death 1 (PD‐1) or programmed cell death‐ligand 1 (PD‐L1) inhibitors, a fibroblast growth factor receptor (FGFR) inhibitor, and an antibody–drug conjugate [3, 4]. Ongoing clinical trials are investigating new treatments [4, 5], some of which require genomic testing to determine treatment eligibility, as is already the case with erdafitinib, an FGFR inhibitor.

Genomic testing for FGFR2, FGFR3, and neurotrophic tyrosine receptor kinase (NTRK) 1–3 alterations are listed in the National Genomic Test Directory for Cancer for use when molecular assessment supports diagnosis or management of urothelial cancer or in line with National Institute for Health and Care Excellence (NICE) recommendations [6]. An FGFR inhibitor is not a first‐line treatment but is mentioned in the NICE guidelines as a treatment for unresectable or mUC with susceptible FGFR3 alterations following at least one line of treatment which includes a PD‐1 or PD‐L1 inhibitor [7]. The European Association of Urology (EAU) 2025 guidelines mention an FGFR inhibitor more broadly as a later line therapy for treating unresectable or mUC, and the European Society for Medical Oncology (ESMO) guidelines for the management of bladder cancer include it as a treatment for relapsed advanced or mUC [3, 8].

There are several barriers to delivering genomic testing for precision medicine in unresectable or mUC. These include the need for extra resources and capacity [9]. Associated costs, data management, and educational requirements of healthcare professionals (HCPs) also require consideration [10]. Genomic heterogeneity both between and within tumour lesions may be another issue [11, 12]. Establishing a standardised testing pathway is desirable to ensure consistency in patient management and optimise delivery of precision oncology.

The objectives of this study were to establish a UK consensus vision of a standardised genomic testing pathway in unresectable or mUC with clearly defined roles and responsibilities for multidisciplinary team (MDT) members and molecular pathology laboratory teams, and to determine how the pathway needs to evolve to accommodate emerging innovations. This consensus is intended to support HCPs and other team members involved in genomic testing in the NHS. A Delphi consensus has been chosen as opinions from a group of experts working in healthcare, rather than individuals, have been shown to be more reliable [13], and another consensus study has already been used to inform development of a testing pathway for oesophago‐gastric cancer care in the UK [14].

## Subjects and Methods

**Fig. 1:**
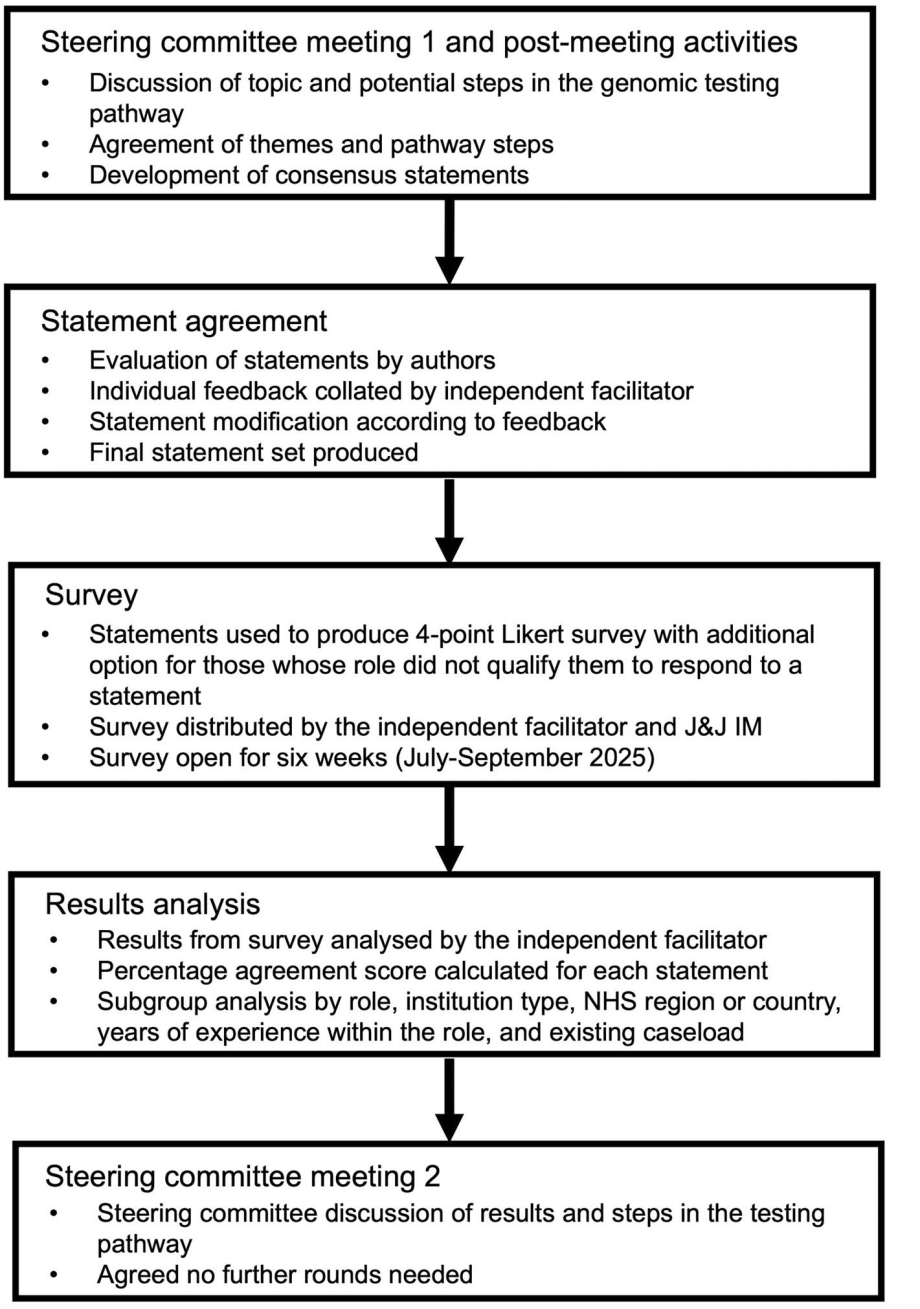
Modified Delphi study design.

A modified Delphi methodology was used (Fig. 1) and reporting in this manuscript follows the ACcurate COnsensus Reporting Document (ACCORD) guidelines [15].

In April 2025, a targeted literature review on genomic testing and precision medicine in patients with unresectable or mUC was conducted. A search for free full texts published in English within the last 5 years was carried out on PubMed. Search terms included but were not limited to ‘urothelial cancer’, ‘bladder cancer’, ‘precision medicine’, and ‘genomic test’. Additional searches were conducted on Google and Google Scholar. Articles were also identified from the reference lists of retrieved papers, and some key background literature was provided by Johnson and Johnson Innovative Medicine (J&J IM).

In May 2025, a Steering Committee (SC) of six HCPs (two histopathologists, two medical oncologists, one clinical oncologist, and one clinical scientist) with expertise in the care and management of patients with UC in the UK met virtually. The findings from the literature review informed the discussion at the first SC meeting, and based on this discussion, seven key topics for consensus were developed:

1. Current and future state of precision medicine for unresectable or mUC in the UK.
2. Step 1 of the pre‐testing pathway – specimen selection, management, and preservation.
3. Step 2 of the pre‐testing pathway – identification, reporting, and storage of the optimal block in case of future genomic testing.
4. Step 1 of the testing pathway – request for genomic tests and sending samples for testing.
5. Step 2 of the testing pathway – genomic testing.
6. Step 3 of the testing pathway – return of genomic testing results.
7. Overcoming barriers and future readiness.

The independent facilitator drafted statements for each topic based on the discussion from the first SC meeting. These were reviewed by all authors, who advised whether each statement should be accepted, reworded, or removed, and could also suggest additional statements. Based on all feedback from the authors, the independent facilitator adjusted the statements and produced the final survey for panellists.

A total of 42 statements were incorporated into a 4‐point Likert survey (‘strongly agree’, ‘tend to agree’, ‘tend to disagree’, ‘strongly disagree’) with an additional option for respondents whose roles did not qualify them to answer. Free‐text fields were available at the end of each topic to allow panellists to provide qualitative feedback on statements they disagreed with. Respondents were also asked to provide demographic information, including role, institution type, NHS region or country, years of experience within the role, and annual caseload of patients with UC.

Eligible panellists were clinical scientists and HCPs with experience conducting molecular testing in or managing patients with UC in the UK. Members of the SC were not permitted to participate in the survey. The SC shared details of relevant contacts within their professional networks, which the independent facilitator compiled into a database and supplemented with additional contacts from desk research. J&J IM also utilised their own database to identify potential respondents.

The survey was electronically distributed by the independent facilitator and J&J IM to the databases of contacts in July 2025 and remained open for 6 weeks, closing in September 2025. The use of their own database and distribution of surveys by J&J IM, as well as addition of contacts from desk research by the independent facilitator, were deviations from the original protocol but were done to aid recruitment, and surveys were only sent to relevant HCPs. All respondents provided consent before completing the survey. The number of individuals invited, and corresponding response rates are unavailable due to the use of combined distribution lists. No incentives were offered; however, respondents could choose to be acknowledged in the study if they wished. Responses were collected by the independent facilitator using Microsoft Forms (Microsoft, Redmond, WA, USA).

To preserve respondent anonymity, only minimal personal data were collected. No personal data were shared with the SC at any point in the study and the identity of individual respondents was not known to either the SC or the independent facilitator. Panellists were asked to provide their professional e‐mail address when completing the survey so that they could be invited to a second round of the survey if required and maintain continuity in the consensus process. However, e‐mail addresses were not linked to individual responses and panellists could choose to complete the survey without providing an e‐mail address.

The consensus threshold was defined a priori as ≥75% agreement, a widely accepted threshold for consensus [16], with stopping criteria met when ≥90% of statements reached consensus. The independent facilitator analysed the completed surveys and calculated an arithmetic agreement score for each statement in Microsoft Excel in September 2025. An overall agreement level was derived from the aggregated responses and subgroup analyses were conducted according to respondent demographics.

During a second meeting held in September 2025, the SC reconvened to review the results of the survey and discuss the development of a genomic testing pathway. One member was unable to attend but provided written feedback after the meeting. The pathway was subsequently ratified by all SC members. As the stopping criteria were met, no further survey rounds were required.

The study did not require ethical approval or registration as no patient‐specific data were collected and only anonymous responses were captured.

## Results

From the initial 45 statements drafted after the first SC meeting, two statements were accepted unchanged, 35 statements were reworded, eight statements were removed, and five were added during reviews. This resulted in 42 statements that were included in the survey for panellists.

Overall, 50 responses to the survey were received from respondents in various roles (15 clinical oncologists, 10 medical oncologists, 10 pathologists, nine urologists, four scientists at a molecular pathology or genomics laboratory, and two nurses; Fig. S1). Most respondents were based in England (*n* = 43 [86%]), with additional representation from Scotland (*n* = 5) and Wales (*n* = 2) (Fig. S2). The majority worked in university or teaching hospitals (*n* = 34 [68%]) and had at least 11 years of experience (*n* = 38 [76%]) (Figs S3 and S4). Among the 39 respondents for whom the question was applicable, the majority managed or saw at least 11 patients with unresectable or mUC each year (*n* = 33 [85%]) (Fig. S5).

**Fig. 2:**
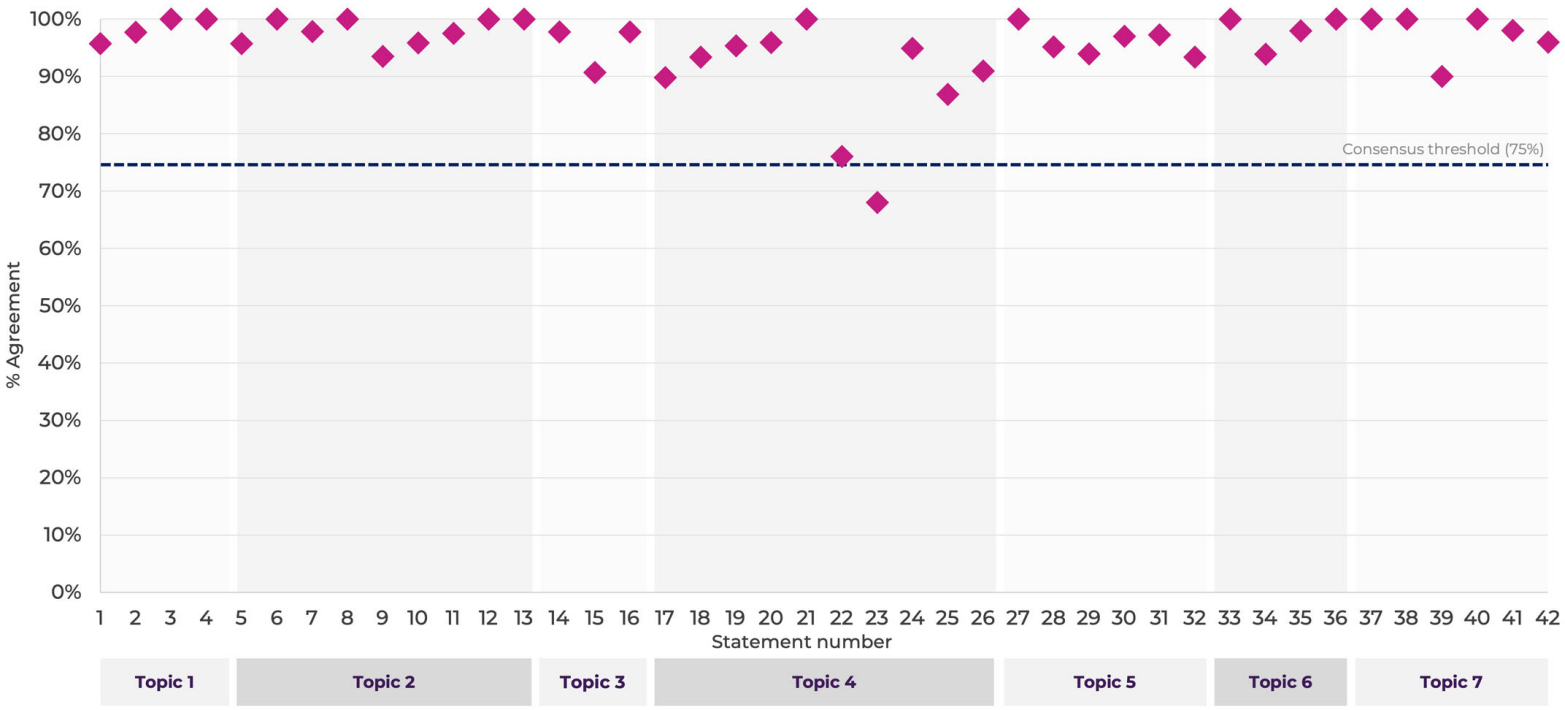
Consensus agreement levels by statement.

Consensus was achieved for 41 out of 42 statements, with 39 statements reaching ≥90% agreement. The one statement that did not reach consensus was part of a paired set (Statement 23 [S23], 68%). Figure 2 and Table 1 show the mean agreement scores from the survey, while summarised qualitative feedback from each topic is provided in Table S1. The full distribution of consensus scores on the 4‐point Likert scale is provided in Fig. S6.

**Table 1:** Defined consensus statements and corresponding levels of agreement.

Statement number	Statement	Agreement score*, %
**Topic 1. Current and future state of precision medicine for unresectable/mUC in the UK**		
S1	The current lack of details in UK guidance regarding the genomic testing pathway for somatic gene alterations in unresectable/mUC contributes to variation in this testing pathway	96 (*n* = 47)
S2	Targeted therapies based on genomic alterations represent a potential treatment option for patients, but, despite reimbursement, usage varies across the UK	98 (*n* = 43)
S3	The UK testing environment needs to be ready to integrate emerging biomarker‐driven therapies into UK clinical pathways to reflect global advancements in care	100 (*n* = 49)
S4	Adequate funding for the GMS is needed to ensure that emerging biomarker‐driven therapies can be integrated into UK clinical pathways	100 (*n* = 47)
**Topic 2. Step 1 of the pre‐testing pathway – specimen selection, management, and preservation**		
S5	Guidance regarding the processing and fixation of different types of tissue (transurethral resection of bladder tumour [TURBT]/cystectomy/metastatic site) is needed to ensure good quality samples for molecular testing	96 (*n* = 47)
S6	To enable genomic testing, the most appropriate specimens should be prioritised for analysis	100 (*n* = 48)
S7	In most cases of advanced UC, the most appropriate specimen for genomic testing will be the most recent evaluable sample, with samples from metastases preferred over samples from primary invasive tumours, and avoiding use of samples from prior non‐muscle‐invasive tumours when possible	98 (*n* = 46)
S8	On some occasions, the most appropriate specimen for genomic testing will be one from TURBT or cystectomy due to the likely greater volume of tissue	100 (*n* = 45)
S9	Circulating tumour DNA testing for genetic aberrations should be considered in patients where tissue testing has failed either due to inadequacy of available tissue or where adequate tissue cannot be obtained	93 (*n* = 46)
S10	The individual performing the resection should consider the adequacy and quality of the sample for future genomic analysis at the time of performing it	96 (*n* = 48)
S11	Specimens should be formalin‐fixed and paraffin‐embedded according to current GCLP	98 (*n* = 40)
S12	The individuals involved in sample fixation should consider the adequacy and quality of the sample for future genomic analysis	100 (*n* = 44)
S13	Timely fixation, paraffin embedding, and keeping the resected specimen at the correct temperature are required to prevent specimen degradation	100 (*n* = 36)
**Topic 3. Step 2 of the pre‐testing pathway – identification, reporting, and storage of the optimal block in case of future genomic testing**		
S14	Where possible, the reporting pathologist should identify and document the best block for future genomic testing	98 (*n* = 45)
S15	The initial histopathology report should state the percentage tumour cellularity, neoplastic cell count, and percentage necrosis in the block which could later be sent for genomic testing	91 (*n* = 43)
S16	Blocks identified as optimal for genomic testing should ideally be stored in an accessible location and be retrievable within 3 days	98 (*n* = 44)
**Topic 4. Step 1 of the testing pathway – request for genomic tests and sending samples for testing**		
S17	The oncologist should communicate with the local pathologist regarding genomic tests needed on a case‐by‐case basis where the test result is not already available	90 (*n* = 49)
S18	FGFR3 tests should be requested reflexively for all patients starting first‐line chemotherapy for advanced UC, ensuring the targeted treatment options are available if a patient progresses	93 (*n* = 45)
S19	FGFR3 tests should be requested for all patients starting or already receiving PD‐1 or PD‐L1 inhibitors as treatment for unresectable/mUC	95 (*n* = 43)
S20	It is advisable to have a clearly defined standard operating procedure in each centre to outline which HCP is responsible for sending the genomic request forms to the molecular pathology laboratory**	96 (*n* = 49)
S21	The pathology team is responsible for removing the block (or requesting the block if they do not have access to it) and sending slides or unmounted tissue to the molecular pathology laboratory for testing	100 (*n* = 44)
S22	Whenever possible, unmounted tissue should be sent to the molecular pathology laboratory** for testing rather than slides	76 (*n* = 25)
S23	Whenever possible, slides (5 × 10 μm) should be sent to the molecular pathology** laboratory for testing rather than unmounted tissue	68 (*n* = 25)
S24	Genomic tests should ideally be conducted on blocks which are <18 months old	95 (*n* = 39)
S25	Where possible, use of blocks older than 6 years should be avoided to prevent test failures	87 (*n* = 38)
S26	A more recent block, conducting a re‐biopsy, or using an appropriate liquid biopsy should be considered before using blocks older than 6 years	91 (*n* = 44)
**Topic 5. Step 2 of the testing pathway – genomic testing**		
S27	Modern and efficient validated methods should be used for DNA extraction in genomic testing of tumour samples from patients with unresectable/mUC	100 (*n* = 43)
S28	NGS should be conducted at a molecular pathology laboratory** for genomic testing of tumour samples from patients with unresectable/mUC	95 (*n* = 41)
S29	For FGFR3 testing, both DNA and RNA panels are needed to identify mutations, gene fusions, and other relevant alterations, ensuring comprehensive results for patients with unresectable/mUC	94 (*n* = 33)
S30	NGS testing can use methods that are not officially CE‐marked, provided they are internally validated and accredited and the testing is conducted in an accredited (UKAS) molecular pathology laboratory** that meets the agreed criteria for exemption from MHRA guidelines on CE marking	97 (*n* = 33)
S31	NTRK testing can be conducted at the time of FGFR3 testing or in patients with no other satisfactory treatment options	97 (*n* = 36)
S32	Genomic testing reports from the molecular pathology laboratory** should have all clinically relevant genomic information summarised at the beginning with indication of biological impact, actionable alterations, and eligibility for specific drugs	93 (*n* = 45)
**Topic 6. Step 3 of the testing pathway – return of genomic testing results**		
S33	All potentially important genetic alterations from the molecular pathology** laboratory should be stored on a national repository to allow later review of any important information not included in the report, such as data relevant to clinical trials or treatment eligibility	100 (*n* = 48)
S34	All centres should ensure that all members of the MDT have immediate access to the genomic testing report once available	94 (*n* = 49)
S35	Genomic biomarker testing results must be available to the responsible clinical team in a timeframe which does not unduly delay patient treatment. Ideally the maximum time between referral for genomic biomarker testing and availability of results should be no >4 weeks	98 (*n* = 48)
S36	Results should be returned from the molecular pathology laboratory within 14 working days of receiving the request and sample	100 (*n* = 49)
**Topic 7. Overcoming barriers and future readiness**		
S37	Ensuring equitable access to genomic testing and biomarker‐informed treatments across all regions of the UK is essential to avoid disparities in care for patients with unresectable/mUC	100 (*n* = 50)
S38	It is vital that HCPs responsible for the care of patients with unresectable/mUC are knowledgeable about genomic biomarker testing and different treatment options for patients to ensure optimal outcomes for patients	100 (*n* = 50)
S39	Training on the optimal fixation of specimens for genomic testing is essential for all relevant staff members	90 (*n* = 50)
S40	As part of Continuing Medical Education, pathologists should be made aware of any biomarker‐driven therapies that are likely to come into routine clinical practice in order to prepare appropriately for implementation	100 (*n* = 50)
S41	Establishing clear frameworks for each step of the genomic pre‐testing and testing pathway can facilitate equitable access and provide centres with the tools to effectively audit their services against quality performance indicators	98 (*n* = 50)
S42	HCPs should monitor rates of testing and identification of clinically important genomic alterations as part of the process to ensure equality of access to high quality care	96 (*n* = 50)

Green shading indicates agreement ≥75%, and red shading indicates agreement <75%.

CE, Conformité Européenne; GCLP, Good Clinical Laboratory Practice; GMS, Genomic Medicine Service; MHRA, Medicines and Healthcare products Regulatory Agency; NGS, next‐generation sequencing; TURBT, transurethral resection of bladder tumour; UKAS, United Kingdom Accreditation Service.

* Percentages were rounded to the nearest whole number prior to assessing whether the threshold for consensus was met.

** Molecular pathology laboratories is the umbrella term used for the relevant laboratories in the UK (i.e., Genomic Laboratory Hubs in England, the All Wales Medical Genomics Service [AWMGS] in Wales, the Scottish Strategic Network for Genomic Medicine [SSNGM] in Scotland, and the Northern Ireland Regional Molecular Diagnostics Service [RMDS] in Northern Ireland) [17, 18].

Sub‐analyses were conducted to assess whether consensus results varied by role, institution type, NHS region or country, years of experience within the role, and existing caseload of patients with UC. There was general alignment between roles, with pathologists less agreeable to some statements (S22, S26, and S32) (Fig. S7). Few other differences by subgroup were observed, and no firm conclusions were drawn due to very small sample sizes within individual groups. Results from subgroup analyses are shown in Figs S7–S11.

## Discussion

### Current and Future State of Precision Medicine for Unresectable or mUC in the UK

The National Genomic Test Directory for Cancer has recently been updated, expanding eligibility for genomic testing among patients with unresectable or mUC [6]. While genomic laboratory hubs in England follow this guidance, similar directives are not yet established in other parts of the UK. HCPs tend to refer to the NICE guidelines [7] rather than to the genomic test directory regarding which patients with unresectable or mUC should undergo genomic testing and when this testing should occur. The NICE guidelines and the National Genomic Test Directory for Cancer both lack details around the genomic testing pathway in unresectable or mUC, including at what point testing should be conducted. This lack of clarity and production of a national genomic test directory in England only may contribute to the varied awareness of availability and differences in uptake of targeted treatments across the UK (S2, 98%).

Adequate funding is also essential to integrate genomic biomarker‐driven therapies into UK clinical pathways (S4, 100%) and to ensure patients receive personalised care, which is a key priority in England's 10‐year health plan and one valued across other parts of the UK [19, 20].

### Pre‐Testing Pathway

#### Step 1 – Specimen Selection, Management, and Preservation

Good‐quality samples are essential for molecular testing; however, poor fixation and prolonged delays compromise sample integrity. There is a recognised need for clearer guidance on the optimal tissue processing and fixation in UC to improve sample quality (S5, 96%).

Regarding selection of the most appropriate specimen, more recent samples with greater tissue volume are preferred (S7, 98%; S8, 100%). Most recent specimens may be from metastatic sites, but samples from bone metastases should be avoided as they may not accurately reflect the tumour's genomic profile and sampling can be challenging [21]. Genomic heterogeneity between the primary tumour and metastases must also be considered as a prospective study reported a 23% discordance in genomic alterations between primary and metastatic sites in UC [11]. The genomic profile of metastases is most relevant.

If suitable tissue is not available, circulating tumour DNA may be an option for genomic testing (S9, 93%), although this is not currently funded in the UK. While most research has focused on its use as a prognostic marker in UC, it holds great potential for guiding therapeutic decisions [22], especially if proven to be cost‐effective.

Although there was unanimous consensus that timely fixation is needed (S13, 100%), a defined window between tissue collection and fixation would be beneficial to ensure consistent sample quality and reliable results for genomic testing. A 48‐h window was suggested by the SC as optimal. At present, many hospitals may not be able to achieve this due to weekend surgeries and laboratory opening hours. Nevertheless, setting such a target could encourage service improvements and underline the need for proper storage conditions (e.g., refrigeration), and adequate use of formalin when the 48‐h period cannot be met. Utilising vacuum packing for tissue preservation is another potential option, as demonstrated in the 100 000 Genomes Project [23].

#### Step 2 – Identification, Reporting, and Storage of the Optimal Block in Case of Future Genomic Testing

The reporting pathologist should select and document the most suitable tissue block for future genomic testing (S14, 98%). Ideally, the histopathology report should state the percentage tumour cellularity, neoplastic cell count, and percentage necrosis in the block (S15, 91%), although this level of detail may be challenging for pathologists due to time constraints. Blocks should be retrievable within 3 days (S16, 98%) to ensure adequate time after the 48‐h period allowed for retrieval of off‐site blocks in service level agreements. The SC noted that even retrieval of nearby blocks can be delayed due to understaffing. Despite potential funding constraints, ensuring there is a staff member whose main responsibility is block retrieval remains a priority.

### Testing Pathway

#### Step 1 – Request for Genomic Tests and Sending Samples for Testing

While S18 (93%) recommends that FGFR3 testing should be requested reflexively for all patients starting first‐line chemotherapy for advanced UC, the authors suggest adjusting to first‐line systemic therapy to reflect the new approval of enfortumab vedotin with pembrolizumab for untreated unresectable or mUC when platinum‐based chemotherapy is suitable [24]. Both reflexive testing and testing for patients starting or already receiving PD‐1 or PD‐L1 inhibitors achieved consensus (S18, 93%; S19, 95%). Genomic testing should therefore be performed early enough in the treatment pathway to ensure that results are available as soon as the patient is eligible for the next line of treatment. There is a small window of opportunity for treatment of many patients with unresectable or mUC, as worsening of performance status with progressive disease often precludes patients from receiving any further treatment.

Reflex testing helps ensure that all patients who may be eligible for FGFR inhibitor therapy are identified and can help prevent delays in treatment escalation. However, the upfront costs associated with genomic testing may present challenges and must be considered. A Latin American consensus on the evaluation and management of patients with metastatic or locally advanced UC reported findings consistent with this Delphi consensus, with 100% agreement that molecular or genomic testing should be performed early, ideally at the time of diagnosis of advanced bladder cancer [25]. These findings highlight the importance of incorporating genomic testing early within the clinical pathway to enable timely identification of patients eligible for targeted therapies and help reduce disparities in access to treatment at a national level. However, not all patients eligible for FGFR inhibitor treatment should receive genomic testing. Individual clinicians should only request testing for patients who they deem fit enough to receive this treatment.

Developing a standard operating procedure within each centre could help clarify who is responsible for sending genomic request forms to the molecular pathology laboratory (S20, 96%). Clearer general guidance may be preferred by some trusts.

Statements 22 and 23 addressed whether unmounted tissue or slides should be sent for testing. S23, which stated a preference for unmounted tissue, did not reach the consensus threshold (68%), but was within 10% of S22 (76%), which stated a preference for slides. This small difference prompted questions for the SC, who noted that guidance on using mounted vs slide samples varies across molecular pathology laboratories. Some respondents may have agreed to both statements as they do not have a preference for slides or unmounted tissue. Qualitative feedback indicated that the choice depends on the laboratory's preference and expertise. Thus, it was agreed by the SC that regarding this choice, local procedures should be followed.

Although newer blocks are preferred for genomic testing over older blocks (S26, 91%), the 6‐year threshold for a suitable block age is not fixed. In the past, this cut‐off may have been more relevant, but with improvements in fixation and sample handling processes, the risk of false negative results is likely reduced. The most recent block should be prioritised, unless it is from bone metastases. Although there was consensus that a more recent block, conducting a re‐biopsy, or using a liquid biopsy should be considered before blocks older than 6 years (S26, 91%), the SC noted that an older block might be preferrable to a re‐biopsy in many cases to avoid the patient undergoing another biopsy.

#### Step 2 – Genomic Testing

One NTRK inhibitor is recommended in NICE technology appraisal guidance and available via the Cancer Drugs Fund in England for treating NTRK fusion‐positive solid tumours if there are no other satisfactory treatments [7]. NTRK testing can be done simultaneously with FGFR3 testing (S31, 97%) and is included in the National Genomic Test Directory for unresectable or mUC in England [6]. Despite this, some trusts in England no longer conduct NTRK testing reflexively due to funding limitations. NTRK could be a more appealing option for funders if results were routinely used to guide treatment decision, which is currently not the case.

The SC also observed that the Genomic Medicine Service in England has not been evaluated against a clear model. Its overall effectiveness is therefore uncertain, and there may be areas that require improvement.

#### Step 3 – Return of Genomic Testing Results

There is currently no national data repository for genomic results. Establishing one would be valuable (S33, 100%) and would require secure data management systems to ensure patient confidentiality [10], as well as ideally having federated access for authorised users. Molecular pathology laboratories often conduct tests beyond those required for immediate patient care to support future research and subsequent patient treatments without the requirement for repeat tests. Audits are needed to assess the effectiveness of this process. If the data are underutilised, further investigations are needed to determine how usage can be optimised. Alternatively, limiting the number of tests and storing smaller amounts of sensitive data should be considered.

Timely access to results by MDT members responsible for clinical decisions is vital (S34, 94%; S35, 98%). In this study, there was consensus that the maximum timeframe between referral for genomic testing and availability of results should be 4 weeks (S35, 98%). This accommodates the maximum of 14 working days required from submission of the request to receipt of results from a molecular pathology laboratory (S36, 100%).

### Overcoming Barriers and Future Readiness

All respondents in this study agreed that ensuring equitable access to genomic testing and biomarker‐informed treatments across all UK nations and regions is important to avoid disparities in care for patients with unresectable or mUC (S37, 100%). However, achieving uniformity is challenging due to variations in resources, infrastructure, and local policies. Ensuring prompt genomic testing requires shared responsibility across the MDT and implementing a standardised genomic testing pathway is a crucial step towards equitable access and optimised use of targeted therapies. The genomic testing pathway described in this manuscript would benefit from a national professional body to support dissemination and standardisation.

### Strengths and Limitations

This Delphi study demonstrated strong agreement, and there was good alignment across different professional roles. All except one statement, due to being part of a pair, reached consensus. Achieving consensus, even for those statements that are seemingly indisputable reinforces their recognition as best practice and provides a foundation for implementation. For example, unanimous agreement on the importance of equitable access underscores its central importance in genomic testing. The 4‐point Likert scale minimised middle option bias, and the additional option for respondents to indicate that their role did not qualify them to respond ensured that only qualified respondents contributed to the score for each statement. Even with this fifth option, the number of respondents for each statement remained sufficient to provide confidence in the consensus reached.

Limitations of this study include less representation of some roles (e.g., nurses) and the majority of respondents being from England (86%), with no respondents from Northern Ireland, potentially limiting generalisability. The high levels of consensus could also suggest that statements were inherently agreeable. However, variability in responses across roles and regions suggest the findings are valid.

### Proposed Genomic Testing Pathway for Unresectable or mUC

Based on the consensus findings and SC discussions, a proposed genomic testing pathway for unresectable or mUC was developed (Table 2) and approved by all authors.

**Table 2:** Pre‐testing and genomic testing pathways for unresectable/mUC.

Pre‐testing pathway	Step 1 Specimen selection, management, and preservation	Which sample should be selected and prioritised for testing?	Select the most suitable sample which is typically the most recent evaluable one with sufficient volume of tissue. Metastatic samples are usually preferred (except bone marrow metastases), though TURBT or cystectomy specimens may be most appropriate due to greater tissue volume.
		Who should ensure the sample is adequate for testing?	The resecting clinician should ensure the sample is adequate for future genomic testing.
		How should the sample be processed?	Follow local guidelines for processing and fixation of different types of tissue (TURBT/cystectomy/metastatic site).
		How should the sample be fixed and stored?	Fix promptly in formalin, preferably within 48 h of tissue collection. Embed in paraffin as per current GCLP. Store under correct conditions.
		What if tissue testing fails?	Consider circulating tumour DNA testing for genetic alterations only if tissue testing has failed.
	Step 2 Identification, reporting, and storage of the optimal block in case of future genomic testing	Who should identify the block for future genomic testing?	The reporting pathologist should identify and document the best block for future genomic testing.
		What details should be reported?	The histopathology report should include tumour cellularity, neoplastic cell count, and percentage necrosis for potential genomic analysis.
		Where should the block be stored?	Optimal blocks should be stored accessibly and be retrievable within 3 days.
Testing pathway *(The time between referral for genomic testing and return of results should not exceed 4 weeks)*	Step 1 Sending the samples for testing	Who coordinates testing when results are not already available?	The oncologist should communicate with the local pathologist regarding genomic tests needed on a case‐by‐case basis.
		When should FGFR3 testing be requested?	Request FGFR3 testing as early as possible in the treatment pathway for unresectable or mUC to ensure targeted therapy options are available if the patient progresses. Ideally, perform reflex testing for all patients with unresectable or mUC starting first‐line systemic therapy.
		Who is responsible for sending requests?	Each centre should have a standard operating procedure or guidance defining which HCP is responsible for sending genomic request forms to the molecular pathology laboratory.
		Who is responsible for removing the block?	The pathology team is responsible for removing the block (or requesting it if they do not have access).
		Which blocks are preferred?	More recent blocks are preferred. For blocks older than 6 years, consider a liquid biopsy. Older blocks may be preferrable to conducing a re‐biopsy (unless clinically necessary).
		How should samples be sent?	Send slides or unmounted tissue according to local guidance.
	Step 2 Genomic testing at the molecular pathology laboratory*	Which testing method should be used?	Testing should be conducted via next‐generation sequencing at the NHS molecular pathology laboratories. CE marking is not required if the laboratory has internal validation and is a UKAS‐accredited molecular pathology laboratory that meets the meeting MHRA criteria for exemption from CE marking.
		Which panels are needed for FGFR3 testing?	Both DNA and RNA panels are needed.
		When should NTRK testing be performed?	Ideally, NTRK testing should be conducted at the same time as FGFR3 testing.
		How should results be reported?	Reports should summarise all clinically relevant genomic information at the beginning, including biological impact, actionable alterations, and eligibility for specific drugs.
	Step 3 Return of genomic testing results	When should results be returned?	Results should be returned by the molecular pathology laboratory within 14 days of receipt of the request and sample.
		How should genomic data be stored?	Data on all potentially important genetic alterations should be stored in secure environments with federated access. Ideally, a national data repository should be established.
		Who should have access to results?	All centres should ensure that MDT members responsible for patient care have immediate access to the genomic testing report once available.

CE, Conformité Européenne; GCLP, Good Clinical Laboratory Practice; MHRA, Medicines and Healthcare products Regulatory Agency; TURBT, Transurethral resection of bladder tumour; UKAS, United Kingdom Accreditation Service.

* Molecular pathology laboratories is the umbrella term used for the relevant laboratories in the UK (i.e., Genomic Laboratory Hubs in England, the All Wales Medical Genomics Service [AWMGS] in Wales, the Scottish Strategic Network for Genomic Medicine [SSNGM] in Scotland, and the Northern Ireland Regional Molecular Diagnostics Service [RMDS] in Northern Ireland).

## Conclusion

The findings from this Delphi consensus study resulted in the generation of a genomic testing pathway for unresectable or mUC including defined roles across the MDT and molecular pathology laboratory team and guidance on timing, methodology, and biomarker selection. This paper includes practical recommendations for integrating genomic testing into the NHS treatment pathway and provides consensus‐based guidance to inform national policy and updates to clinical practice. This should support alignment across NHS trusts and future revisions to UK guidelines for unresectable or mUC.

## Funding

The study was initiated and funded by Janssen‐Cilag Limited, a Johnson and Johnson company. All authors received funding from Janssen‐Cilag Limited while undertaking this study. Janssen‐Cilag Limited commissioned Triducive Partners Limited to facilitate the project and analyse the responses to the consensus statements in line with the Delphi methodology. Two authors of the manuscript are employees of Janssen‐Cilag Limited.

## Disclosure of Interests

The authors state the following conflicts of interest: Amarnath Challapalli received payment for involvement in this project which was sponsored by Janssen‐Cilag Limited. The payment was made to himself. He received honoraria (paid to himself) for a speaker meeting on erdafitinib at the UK Oncology Forum organised by Janssen. Travel and accommodation were provided for him by Janssen at their prostate summit. Simran Gill has attended occasional congresses supported by her employer (J&J), but these were not related specifically to this manuscript. She holds stocks in J&J and is a current employee (Medical Lead, Bladder Cancer) at J&J. Her salary is paid by J&J. Michael Hubank was paid as a SC member for this project funded by Janssen‐Cilag Limited. He works at the Royal Marsden which has a commercial partnership with circulating tumour DNA test providers, Guardant Health. He was paid for meeting presentations by Guardant Health and AstraZeneca. He was paid for webinars by Servier and Diaceutics. He was paid for advisory boards by Guardant Health, AstraZeneca, J&J, and Seagen. He was paid for a consultation on circulating tumour cells by Alira Health. Syed A Hussain received payment for involvement in this project which was funded by Janssen‐Cilag Limited. He has received grants or contracts from Roche. He has received payment or honoraria for lecture, presentations, speakers’ bureaus, manuscript writing or educational events for Merck, Janssen, Roche, AstraZeneca, MSD, Bristol Myers Squibb, GSK, Astellas, and Pfizer. He has received support for attending meetings and/or travel from Janssen, Merck, and MSD. Robert Jones received payment for involvement in this project which was funded by Janssen‐Cilag Limited. He has received grants from Astellas, Clovis, Exelixis, Bayer, and Roche. He has received advisory board fees from Janssen, Astellas, Bayer, Novartis, Pfizer, Merck Serono, MSD, Roche, Ipsen, and Bristol Myers Squibb. He has received lecture honoraria from Astellas, Janssen, Bayer, Pfizer, Merck Serono, MSD, Roche, Ipsen, and Bristol Myers Squibb. He has received support for conference attendance from Bayer and Janssen. He has participated on a data safety monitoring board or advisory board for Roche/Genentech. Hannah Markham received a payment direct to herself from Janssen‐Cilag Limited for involvement in this project which was funded by Janssen‐Cilag Limited. Philippe Tanière received payments from Janssen‐Cilag Limited for involvement in this study which was sponsored by J&J IM. The payments were made to his own account and declared to the trust (Caldicott). He received speaker fees from Agilent, Astellas, AstraZeneca, Biocartis, Bristol Myers Squibb, Diaceutics, Eli Lilly, Janssen, Merck Serono, MSD, Novartis, Pfizer, Roche, and Takeda. Payments were made to his own account and declared to the trust (Caldicott). He was supported by Biocartis in 2024 to attend the ESMO meeting. He was supported by Astellas in 2024 to attend the European Congress of Pathology meeting. He received fees for participation in advisory boards from Agilent, Astellas, AstraZeneca, Biocartis, Bristol Myers Squibb, Diaceutics, Eli Lilly, Janssen, Merck Serono, MSD, Novartis, Pfizer, Roche, and Takeda. Payments were made to his own account and declared to the trust (Caldicott). Simon Wyatt is a current employee (Senior Medical Advisor, Bladder Cancer) at J&J. His salary is paid by J&J. He is supported by his employer for occasional congress attendance as part of his role. This is not related specifically to this manuscript.

## Author Contributions

All authors developed and/or reviewed the initial statements and read and approved the final manuscript. Amarnath Challapalli, Michael Hubank, Syed A Hussain, Robert Jones, Hannah Markham, and Philippe Tanière also contributed to the interpretation and discussion of results.

## Ethics Statement

As this study only collected the anonymous opinions of clinical scientists and HCPs and no patient‐specific data were captured, ethical approval was not sought.

## Supporting information


**Fig. S1.** Number of respondents per role.
**Fig. S2.** Number of respondents by region/country.
**Fig. S3.** Number of respondents by type of institution.
**Fig. S4.** Number of respondents by years of experience.
**Fig. S5.** Number of respondents by annual caseload of patients with UC.
**Fig. S6.** Percentages of agreement level by statement.
**Fig. S7.** Consensus agreement levels for each statement by respondent role.
**Fig. S8.** Consensus agreement levels for each statement by type of institution.
**Fig. S9.** Consensus agreement levels for each statement by region/country.
**Fig. S10.** Consensus agreement levels for each statement by years of experience.
**Fig. S11.** Consensus agreement levels for each statement by annual caseload of patients with UC; not applicable for nine respondents.
**Table S1.** Summary of qualitative feedback by topic.

## Data Availability

The datasets used and/or analysed during the current study are available from the corresponding author on reasonable request.
