# Supplementary material for: A Delphi consensus for implementing genomic testing in unresectable or metastatic urothelial cancer
Source: BJU Int. 2026 Apr 27;138(1):144–55. doi: 10.1111/bju.70278 (PMC13244585; doi:10.1111/bju.70278)
Supplement: Supplementary file 1 — Fig. S1. Number of respondents per role. Fig. S2. Number of respondents by region/country. Fig. S3. Number of respondents by type of institution. Fig. S4. Number of respondents by years of experience. Fig. S5. Number of respondents by annual caseload of patients with UC. Fig. S6. Percentages of agreement level by statement. Fig. S7. Consensus agreement levels for each statement by respondent role. Fig. S8. Consensus agreement levels for each statement by type of institution. Fig. S9. Consensus agreement levels for each statement by region/country. Fig. S10. Consensus agreement levels for each statement by years of experience. Fig. S11. Consensus agreement levels for each statement by annual caseload of patients with UC; not applicable for nine respondents. Table S1. Summary of qualitative feedback by topic. [file BJU-138-144-s001.docx]

**SUPPLEMENTARY INFORMATION**

**A Delphi consensus for implementing genomic testing in unresectable or metastatic urothelial cancer**

Syed A. Hussain^1^, Amarnath Challapalli^2^, Simran Gill^3^, Michael Hubank^4^, Hannah Markham^5^, Philippe Tanière^6^, Simon Wyatt^3^, Robert Jones.^7^

1. Division of Clinical Medicine, School of Medicine and Population Health, University of Sheffield and Sheffield Teaching Hospitals, Sheffield. UK.
2. Bristol Cancer Institute, Bristol. UK.
3. Johnson & Johnson Innovative Medicine, High Wycombe, UK.
4. Royal Marsden Hospital, London, UK.
5. University Hospital Southampton, Southampton. UK.
6. Queen Elizabeth Hospital, Birmingham. UK.
7. University of Glasgow, Beatson West of Scotland Cancer Centre, Glasgow. UK

**CORRESPONDING AUTHOR:**

Professor Syed A Hussain

Division of Clinical Medicine, School of Medicine and Population Health, University of Sheffield and Sheffield Teaching Hospitals, Sheffield. UK.

syed.hussain@sheffield.ac.uk

**Figure S1.** Number of respondents per role

**Figure S2.** Number of respondents by region/country

**Figure S3.** Number of respondents by type of institution

**Figure S4.** Number of respondents by years of experience

**Figure S5.** Number of respondents by annual caseload of patients with urothelial cancer

**Table S1.** Summary of qualitative feedback by topic

| **Topic** | **Qualitative feedback summary** |
| --- | --- |
| **1. Current and future state of precision medicine for unresectable/mUC in the UK** | - The National Genomic Test Directory clearly indicates available tests, and stakeholders understand these are delivered by Genomic Laboratory Hubs (GLHs). - Statement 4 does not apply to Scotland where the process is different (arguably more flexible and responsive). |
| **2. Step 1 of the pre-testing pathway – Specimen selection, management, and preservation** | - **Sample quality and type:** Fresh/frozen tissue is preferred for genomic testing, but formalin-fixed paraffin-embedded (FFPE) is widely used and improvements in extraction make it acceptable; guidelines are needed to optimise sample handling and processing. - **Clinical practicality:** The primary aim of transurethral resection of bladder tumour (TURBT)/cystectomy remains diagnosis and tumour clearance; sample adequacy for genomic testing should be considered without delaying treatment; recent invasive samples are preferred. - **Alternative approaches and reporting:** Circulating deoxyribonucleic acid (ctDNA) or urine deoxyribonucleic acid (DNA) may be options when tissue is inadequate, though availability is limited; pathology reports should be structured to support future genomic testing. |
| **3. Step 2 of the pre-testing pathway – identification, reporting, and storage of the optimal block in case of future genomic testing** | - **Workflow and resources:** Rapid turnaround and timely block retrieval are important, but National Health Service (NHS) pressures and current pathology resourcing limit strict timelines; efficient workflows and flexible detail in block handling are needed. - **Expertise and testing capacity:** Trained molecular pathology scientists can perform many tasks effectively; circulating tumour cell testing is not standard of care or reimbursed outside clinical trials. |
| **4. Step 1 of the testing pathway – request for genomic tests and sending samples for testing** | - **Older blocks vs. re-biopsy:** Older blocks can often be used successfully; re-biopsy should be reserved for clinically necessary cases. - **Reflex/routine testing:** Routine panels for actionable alterations are preferred to avoid ad hoc requests, though not all patients need upfront fibroblast growth factor receptor 3 (FGFR3) testing. - **Sample handling:** Choice of slides vs. unmounted tissue depends on tumour content and lab expertise; blocks should go to centres with the right skills. |
| **5. Step 2 of the testing pathway – genomic testing** | - **Reporting content and format:** Reports should focus on actionable genomic alterations and therapy classes rather than specific drug names, with flexibility in where information is presented (e.g. summary at the end). Reports should remain relevant over time, separating test results from treatment guidance. - **Testing capacity and efficiency:** The current seven genomic hubs in England are insufficient; local testing with in-house next-generation sequencing (NGS) could improve turnaround times and efficiency, avoiding delays from shipping blocks to GLHs. - **NGS strategy and prioritisation:** Targeted NGS panels are sufficient for identifying relevant alterations; more complex sequencing (exome, ribonucleic acid sequencing [RNAseq]) may be reserved for future use. Conformité Européenne (CE)-marked tests or UK equivalents are acceptable; testing should be balanced against resource priorities (e.g. neurotrophic tyrosine receptor kinase [NTRK] testing only if it does not divert resources). |
| **6. Step 3 of the testing pathway – return of genomic testing results** | - **Reporting pathway and oversight:** Results should ideally be integrated via pathologists rather than sent directly to clinical teams to catch errors (e.g. contamination) and ensure accuracy, though this should not introduce delays. - **Multidisciplinary team (MDT) access and patient data:** Genetic results should be accessible to MDTs to stimulate engagement, but not all members need immediate access; patient consent and confidentiality must be respected, especially for those not undergoing further therapy. - **Turnaround, databases, and efficiency:** Aiming for rapid turnaround (e.g. 7 days) is desirable; a national database could track trends and variation, but funding, cost-effectiveness, and batching practices need to be considered. |
| **7. Overcoming barriers and future readiness** | - **Training on the optimal fixation:** Not relevant to all staff members. - **Monitoring and relevance:** Healthcare professionals (HCPs) may not need to track testing rates themselves; the Genomic Medicine Service (GMS) can monitor these, while HCPs should focus on whether results guide treatment and patients receive appropriate therapies. |

CE=Conformité Européenne; ctDNA=Circulating DNA; DNA=Deoxyribonucleic acid; FFPE=Formalin-fixed paraffin embedded; FGFR3=Fibroblast growth factor receptor 3; GLH=Genomic laboratory hub; GMS=Genomic Medicine Service; HCP=Healthcare professional; NHS=National Health Service; NTRK=Neurotrophic tyrosine receptor kinase; MDT=Multidisciplinary team; RNAseq= Ribonucleic acid sequencing; TURBT=Transurethral resection of bladder tumour.

**Figure S6.** Percentages of agreement level by statement

**Figure S7.** Consensus agreement levels for each statement by respondent role

**Figure S8.** Consensus agreement levels for each statement by type of institution

**Figure S9.** Consensus agreement levels for each statement by region/country

**Figure S10.** Consensus agreement levels for each statement by years of experience

**Figure S11.** Consensus agreement levels for each statement by annual caseload of patients with urothelial cancer; not applicable for nine respondents
